# Mechanisms of Change in Digital Health Interventions for Mental Disorders in Youth: Systematic Review

**DOI:** 10.2196/29742

**Published:** 2021-11-26

**Authors:** Matthias Domhardt, Sophie Engler, Hannah Nowak, Arne Lutsch, Amit Baumel, Harald Baumeister

**Affiliations:** 1 Department of Clinical Psychology and Psychotherapy Ulm University Ulm Germany; 2 Department of Community Mental Health University of Haifa Haifa Israel

**Keywords:** children and adolescents, mental disorders, mediator, mechanisms of change, digital health intervention, psychotherapy, mobile phone

## Abstract

**Background:**

Digital health interventions (DHIs) are efficacious for several mental disorders in youth; however, integrated, evidence-based knowledge about the mechanisms of change in these interventions is lacking.

**Objective:**

This systematic review aims to comprehensively evaluate studies on mediators and mechanisms of change in different DHIs for common mental disorders in children and adolescents.

**Methods:**

A systematic literature search of the electronic databases Cochrane Central Register of Controlled Trials, Embase, MEDLINE, and PsycINFO was conducted, complemented by backward and forward searches. Two independent reviewers selected studies for inclusion, extracted the data, and rated the methodological quality of eligible studies (ie, risk of bias and 8 quality criteria for process research).

**Results:**

A total of 25 studies that have evaluated 39 potential mediators were included in this review. Cognitive mediators were the largest group of examined intervening variables, followed by a broad range of emotional and affective, interpersonal, parenting behavior, and other mediators. The mediator categories with the highest percentages of significant intervening variables were the groups of affective mediators (4/4, 100%) and combined cognitive mediators (13/19, 68%). Although more than three-quarters of the eligible studies met 5 or more quality criteria, causal conclusions have been widely precluded.

**Conclusions:**

The findings of this review might guide the empirically informed advancement of DHIs, contributing to improved intervention outcomes, and the discussion of methodological recommendations for process research might facilitate mediation studies with more pertinent designs, allowing for conclusions with higher causal certainty in the future.

## Introduction

### Background

Mental disorders in children and adolescents are common, with prevalence rates ranging from 10% to 20% worldwide [1]. These disorders contribute substantially to the global burden of disease in youth [2], and about half of all mental disorders across the life span have their onset in adolescence [3]. Hence, early psychotherapeutic interventions are essential to counteract the risk of chronification and prevent possible negative long-term effects [1]. However, a substantial proportion of children and adolescents with mental disorders do not receive adequate psychotherapeutic or psychosocial care [4-6] owing to different individual and structural barriers to treatment uptake [7]. Furthermore, the availability of mental health care is often insufficient to adequately meet treatment demands, particularly in rural regions [8] and low-income countries [9].

Digital health interventions (DHIs), such as internet- and mobile-based interventions with a psychotherapeutic focus (DHI_PSY_), offer the possibility of addressing some barriers to treatment uptake and might contribute to extending mental health care, given their various presumed advantages, such as possible cost- and time-efficient use, independence from spatial and temporal circumstances, potential anonymity, high degrees of flexibility, and autonomy for users. These assets may be especially important during the COVID-19 pandemic and allow for continued mental health care despite contact restrictions and physical distancing [10]. Furthermore, in view of the fact that youth are particular familiar with digital devices (so-called *digital natives*; Children in a digital world [11]), the use of DHIs might be especially appealing to this younger age group [12]. DHIs can be distinguished and characterized based on their theoretical basis, the type of technical implementation, the area of application, and the extent of accompanying human support [13,14]. The type and the intensity of *guidance* in DHIs can vary on the continuum from (1) pure self-help interventions without any human support (so-called *unguided interventions*) to (2) interventions with some support (*guided interventions*), to (3) videoconference-based psychotherapy with the internet as the sole communication medium between therapists and patients [15].

The efficacy of DHI_PSY_ for some common mental disorders in children and adolescents has been established using meta-analyses [12,16-19]. For example, Vigerland et al [19] evaluated the efficacy of internet-based cognitive behavioral therapy for a range of mental disorders, including anxiety, depression, behavioral problems, obsessive-compulsive disorder, and some somatic disorders such as chronic pain and insomnia. This meta-analysis revealed a moderate, aggregated effect size favoring internet-based cognitive behavioral therapy over waitlist (g=0.62, 95% CI 0.41-0.84; *P*<.001). In contrast, Hollis et al [20] appraised the evidence on the efficacy of DHI_PSY_ for other mental disorders, including attention-deficit/hyperactivity disorder, autism, psychotic disorders, and eating disorders in their review as uncertain and necessitating future research regarding moderators of intervention effects. Current empirical knowledge suggests that older children and adolescents benefit more from DHI_PSY_ than younger children [12,20]. In addition, the well-established finding that guided interventions are more efficacious than unguided interventions in adults [21,22] also seems to apply for DHI_PSY_ in youth [12,20]. Complementing the evidence base and representing another major area of application, DHIs with a focus on health promotion (DHI_HP_), for example, on alcohol consumption or other lifestyle and health behaviors, revealed a considerably smaller effect size (Cohen *d*=0.14, 95% CI 0.00-0.27) [23] when compared with DHI_PSY_ with a genuine psychotherapeutic foundation such as cognitive behavioral therapy (g=0.72, 95% CI 0.55-0.90; *P*<.001) [16].

Given this rather heterogeneous body of research regarding the efficacy and effectiveness of DHIs, comprising various interventions with different theoretical orientations, foci, and delivery modes, for mental health issues in children and adolescents, it seems both timely and worthwhile to investigate the presumed working mechanisms in these technology-delivered interventions. This is because evidence-based knowledge on the mediators and mechanisms of change (specific for different approaches of DHI_PSY_ and DHI_HP_) can inform intervention development and mental health care practices, illustrating pathways to more powerful intervention packages and improved outcomes [24-26]. The first step in understanding the underlying processes in DHIs is to analyze the mediators. A mediator is an intervening variable that can explain the statistical relationship between an independent variable (eg, a DHI) and a dependent variable (eg, a symptom change) [25], and can thereby potentially point to a mechanism through which an intervention achieves its effects. Although various methods for statistical mediation analysis are available (eg, MacKinnon et al [27]), comprising different approaches such as latent growth curve modeling [28] or structural equation modeling [29], the seminal approach of Baron and Kenny [30] is still one of the most applied procedures to evaluate the intervening variable effect of a potential mediator, despite having received criticism with regard to some limitations, such as low statistical power, difficulties in the assessment of multiple mediators, or quantification of the mediation effect magnitude [24]. Although statistical mediation may be established either with the so-called causal-steps approach by Baron and Kenny [30] or by more recent methods correcting some of its limitations (eg, Kraemer et al [31]), it is important to consider that mediators might be identical to a mechanism of change (ie, the actual process responsible for change), but might also be a proxy for 1 or more other variables that do not explain the hypothesized mechanism [25]. Thus, to determine the degree of validity that a mediator is actually representative of for being considered a true change mechanism, Kazdin proposed several quality criteria for psychotherapy process research [25] that can be consulted when assessing the scope and justification of causal inferences: (1) Strong association (among treatment, mediator, and outcome), (2) specificity (a mediator accounts for the indirect effect of treatment on outcome), (3) consistency (the association must be replicable), (4) experimental manipulation (use of either a randomized controlled trial [RCT] design where the intervention variable is manipulated or an experimental design where the mediator itself is directly manipulated), (5) timeline or temporality (the intervention must lead to changes in the mediator, which must temporally precede changes in the outcome), (6) gradient (ie, a dose-response relationship: greater activation of the mediator is associated with greater change in the outcome), and (7) plausibility or coherence (the proposed mediator must be embedded in a plausible theoretical framework).

The evidence base for the mediators and mechanisms of change in conventional face-to-face psychotherapies for children and adolescents is scarce, and only a few studies have been designed to investigate the therapeutic processes in these interventions [32]. For example, 2 systematic reviews dedicated to evaluating the mechanisms of change found that only a small number of eligible primary studies actually conducted mediation analyses, with only 9% (6/67) [33] and 17% (8/46) of included clinical trials [34] attempting to evaluate mediation effects. In addition, Schmidt and Schimmelmann [35] reviewed the empirical literature on mediators in psychotherapeutic interventions for common mental disorders in youth and concluded that most eligible studies evaluated mediators referring to the parent-child interaction (eg, family cohesion and parental support), next to mediators within the patient (eg, self-efficacy, motivation, coping, interpersonal skills, as well as changes in dysfunctional cognitions and negative emotions) and characteristics of the intervention (eg, duration of treatment, number of patient-therapist contacts, and application of specific intervention techniques). The included studies revealed inconsistent patterns of mediation effects related to therapist factors, such as flexibility, adherence to treatment, or therapeutic alliance [35]. Moreover, as central conceptual and methodological requirements for mediation analyses were often not met by studies in this review, causal inferences were widely precluded, necessitating future process research with higher methodological rigor [35].

### Objectives

Although research on DHIs is a fast growing field [36] and might also offer intriguing opportunities for psychotherapeutic process research [24,37], we are not aware of any systematic review of the mediators and mechanisms of change in DHI_PSY_ and DHI_HP_ for common mental disorders in youth published to this point. Therefore, this study aimed to: (1) systematically review the current state of research on mediators and mechanisms of change in various DHIs for mental disorders in children and adolescents, (2) identify mediators and potential mechanisms of change in these interventions, and (3) evaluate the methodological quality of eligible mediation studies according to the quality criteria for process research mentioned above.

## Methods

This systematic review was reported in accordance with the PRISMA (Preferred Reporting Items for Systematic Reviews and Meta-Analyses) guidelines [38] and was a priori registered with the Open Science Framework [39].

### Eligibility Criteria

Studies were eligible for inclusion if they fulfilled the following criteria: (1) participants were children (0-13 years) or adolescents (14-21 years) diagnosed with a mental disorder or exhibited clinically relevant symptoms of a mental disorder; (2) in the case of mixed samples with adolescents and young adults, the mean age of the sample was not above 21 years; (3) the interventions were designed for children or adolescents, or for parents of children or adolescents fulfilling the first criterion; (4) the diagnosis of mental disorders was based on the diagnostic and statistical manual of mental disorders, or the International Classification of Diseases criteria and was assessed with a validated and standardized clinical interview, or a standardized self-report instrument, or standardized ratings by significant others (eg, parents, teachers, clinicians); (5) samples of mixed or comorbid mental disorders were included; (6) studies with different recruitment strategies were eligible; (7) interventions with different theoretical orientations were eligible; (8) the intervention was predominantly delivered through the internet (eg, via web browsers or mobile or smartphone apps); (9) interventions with different degrees of human guidance and completely self-guided interventions were eligible for inclusion; (10) different active and passive control groups (CGs) were included; (11) changes in the symptoms and (12) mediators were reported; (13) at least one mediation analysis was performed; (14) studies were RCTs or secondary analyses of RCTs published in a peer reviewed journal in English language.

### Systematic Literature Search and Study Selection

The search strategy was 3-fold. First, systematic literature searches were conducted in the Cochrane Central Register of Controlled Trials, Embase, MEDLINE (ie, Ovid MEDLINE, Ovid MEDLINE Epub Ahead of Print, Ovid MEDLINE In-Process and Other Non-Indexed Citations, Ovid MEDLINE Daily Update), and PsycINFO databases from database inception until May 30, 2020. The search strings were built on established prior search strings [24,40] further adapted to specifically meet the research questions at hand and modified for each database in Ovid (for details on all search strings, see Multimedia Appendix 1). Second, the reference lists of all eligible studies and other relevant reviews were manually searched to identify further studies that met our inclusion criteria (ie, backward searches). Third, a citation-search (ie, forward search) was conducted in the Web of Science database.

**Figure 1:**
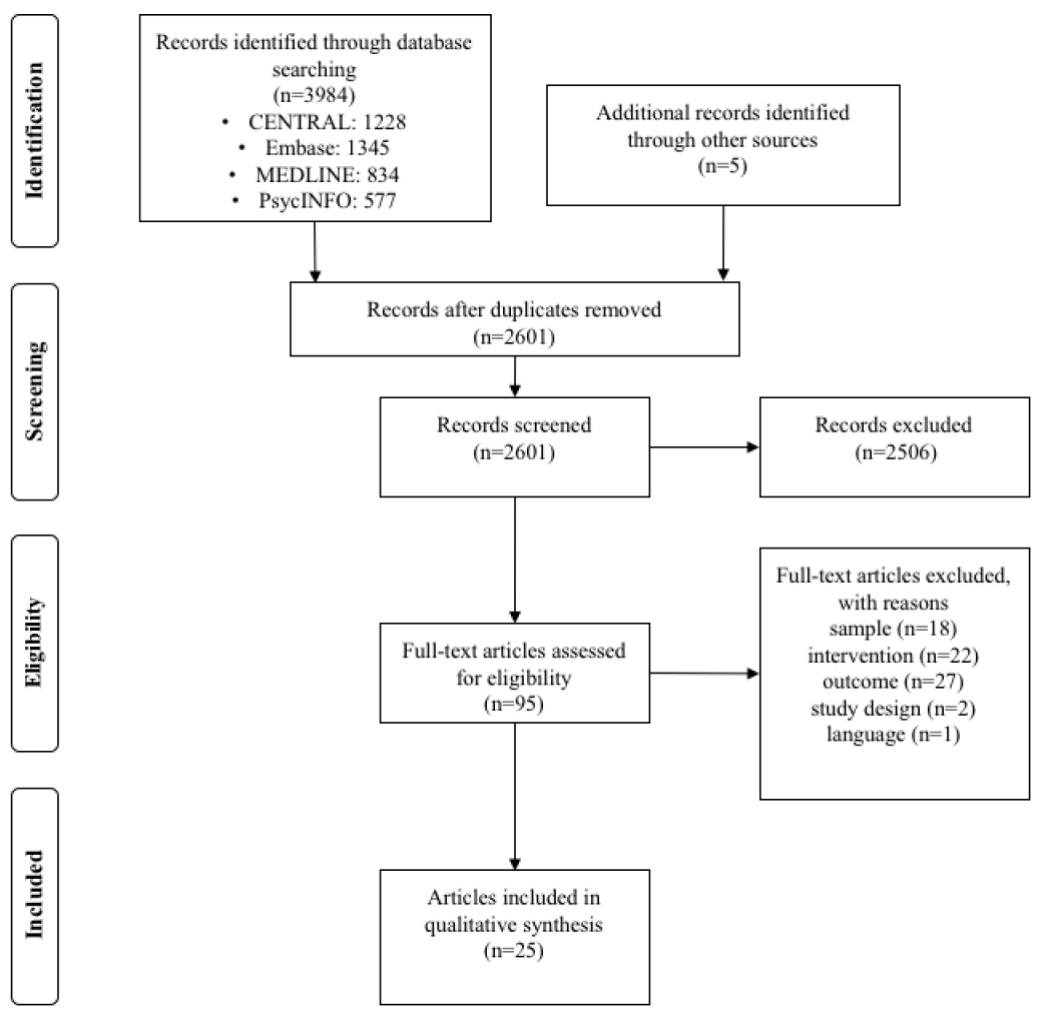
PRISMA (Preferred Reporting Items for Systematic Reviews and Meta-Analyses) flowchart.

Study selection was conducted with the support of a software tool for systematic reviews [41]. Duplicates were detected automatically by the software or were manually removed. First, 1 reviewer (SE) screened titles and abstracts of all the remaining studies and discarded irrelevant articles. Second, the full texts of all potentially relevant articles were screened in terms of the aforementioned eligibility criteria independently by 2 reviewers (SE and HN). Disagreements were resolved by consultation with a third reviewer (MD). The full process of the systematic literature search and study selection is displayed in the PRISMA flowchart of Figure 1.

### Data Extraction

Two independent reviewers (SE and HN) extracted the following data: study information items (name of first author, country, and year), sample information (sample size and age), intervention characteristics (theoretical orientation, number of modules, and duration of intervention), information about control conditions, and information about outcomes (mediator type, instrument, and statistical analysis of mediation). Authors were contacted via email in case of missing information essential for decisions on study selection and the application of the systematic review.

### Categorization of Studies

The included studies were divided into 2 categories: studies evaluating interventions with a psychotherapeutic focus (ie, DHI_PSY_) and studies evaluating interventions with a focus on health promotion (comprising interventions targeting health behavior, lifestyle, and behavior change interventions for the purpose of primary and secondary prevention; ie, DHI_HP_). Of note, interventions with rehabilitation or tertiary prevention focus were not included.

### Quality Assessment

#### Risk-of-Bias Assessment

The methodological quality of the included studies was assessed independently by 2 reviewers (SE and HN) using the Cochrane risk-of-bias (RoB) tool for randomized trials (version 2, RoB2; [42]) on 5 domains: (1) bias arising from the randomization process, (2) bias due to deviations from intended interventions, (3) bias due to missing outcome data, (4) bias in measurement of the outcomes, and (5) bias in selection of the reported result. The included studies were rated as having *low*, *unclear*, or *high RoB* in each domain [42]. Interrater reliability was calculated using the Cohen κ in RStudio (version 1.2.1335; [43]).

#### Quality Criteria for Process Research and Approximating Causality

The included studies were rated by 2 independent reviewers (SE and HN). The rating system was based on Kazdin’s [25] initial criteria to approach causality, modified by Domhardt et al [24] and Lemmens et al [44]. To meet the respective criteria, studies had to: (1) use an appropriate RCT design, (2) include a CG, (3) report a theoretical foundation for mediators, (4) have a minimum sample size of 40 participants per group, (5) examine multiple mediators within 1 study, (6) assess temporality (3 or more assessments of the mediator variables and outcomes), (7) experimentally manipulate the mediator, and (8) reveal a strong statistical association among intervention, mediator, and outcome (operationalized as statistical significance of *P*<.05, as suggested by Moreno-Peral et al [45]). All criteria were rated as fulfilled or not fulfilled. In accordance with prior research [24,44], criteria specificity, consistency, and gradient were not assessed, as they are not meaningfully applicable in single studies (consistency) or are too exclusive for some therapeutic processes (gradient and specificity).

## Results

### Study Characteristics

Altogether, data from 25 (*k*=25) publications were analyzed [46-70]. Specifically, 52% (13/25) of the studies [46,47,49,54,56,58-60,64-67,69] evaluated DHI_PSY_ and 48% (12/25) of the studies evaluated DHI_HP_ [48,50-53,55,57,61-63,68,70]. In the studies, a total of 4884 participants were randomized. Studies on DHI_PSY_ accounted for 43.2% (2110/4884) of participants, and studies on DHI_HP_ accounted for 56.8% (2774/4884) of participants. The overall sample sizes varied from 51 [59] to 818 [63]. The median publication year was 2014 (2002 [48] to 2020 [49]). Most studies were conducted in the United States (17/25, 68%), and most of the study participants were female (2985/4884, 61.18%). In 1 study, no information was provided regarding the distribution of sex [54]. The mean age of participants was 18.49 (SD 2.01) years. The lowest mean age was 5.4 (SD 2.2) years [67], and the highest was 21.02 (SD 2.16) years [70]. Participants were younger in studies evaluating DHI_PSY_ compared with participants in studies evaluating DHI_HP_ (17.11 vs 19.67 years). The exact information on the average age of the final sample was not provided in 4 studies [54,56,63,68]. Most interventions took place without parental involvement and were directed at the youth themselves (20/25, 80%). In 20% (5/25) of the studies, interventions for children [54,67] as well as interventions for both children and adolescents [46,65] and for adolescents only [64] were evaluated with the involvement of parents (including intervention components for parents or parent training). Demographic information about the parents who participated was provided in 16% (4/25) of the studies [46,54,65,67]. Study participants were recruited predominantly from educational or health-related settings (15/25, 60%). In 32% (8/25) of the studies, participants were made aware of the study through both web-based advertising (including social media and websites) and conventional advertising (including letters and flyers). To identify potential participants, one study (1/25, 4%) used data from a mass web-based survey [59]. Information on recruitment strategy could not be identified in 4% (1/25) of the studies [68]. Across studies, the average study dropout rate accounted for 18% (range 0% [62] to 39.3% [54]). In 4% (1/25) of the studies, the dropout rate was not reported [46].

The interventions were directed toward a broad range of mental health problems, including risky drinking behavior (including risky or heavy drinking and binge drinking; 11/25, 44%), depressive disorders (5/25, 20%), anxiety disorders (including separation anxiety, generalized anxiety disorder, social phobia, and specific phobia; 3/25, 12%), behavioral problems (2/25, 8%), and insomnia (1/25, 4%). Furthermore, 12% (3/25) of the studies evaluated interventions that addressed multiple disorders simultaneously (ie, transdiagnostic interventions). De Bruin et al [47] addressed transdiagnostic *psychopathological abnormalities* (including affective, anxiety, and somatic problems; problems concerning attention-deficit/hyperactivity disorder; oppositional defiant behavior; and conduct problems). Levin et al [60] addressed *psychological problems* such as depressive disorders, generalized anxiety disorder, social phobia, alcohol consumption, academic worries, worries concerning eating, hostility, and negative stress. Another intervention addressed depression, anxiety, and stress [56]. Of note, there were 2 interventions evaluated and applied in more than 1 study (the *e-CHUG* interventions in 4 studies; and the *BRAVE* interventions in 2 studies), but the investigated mediators differed in all studies; thus, these publications were regarded as distinct entities and single studies (see Multimedia Appendix 2 [46-70] for details).

Internet-based interventions were evaluated in 92% (12/13) of the studies on DHI_PSY_. Most interventions were based on the components of cognitive behavioral therapy [46,47,49,59,66]. In addition, relaxation strategies, such as progressive muscle relaxation and autogenic training [49], and elements of social learning theory [54] were deployed. Further interventions were based on the acceptance and commitment therapy [60], components of systemic family therapy, problem solving and communication training, cognitive restructuring, and alternative family roles [64]. One intervention [56] was based on the *temporal model of control*. In 23% (3/13) of the studies, precise information on the theoretical orientation and background of the intervention was not available [58,67,69]. Moreover, 7% (1/13) of the studies evaluated a mobile-based intervention based on a self-monitoring program [58].

All DHI_HP_ studies were internet-based and included a feedback component (12/12, 100%). The so-called *e-CHUG* tool was evaluated in 33% (4/12) of the studies [51,57,62,68]. Feedback was provided via email [48,53] or in person using a motivational interviewing approach [51,52]. In addition to the feedback components, cognitive components such as expressive writing [70] and retrieval of remembered information from feedback were evaluated [57]. A detailed overview of the study characteristics is provided in Multimedia Appendix 2.

### Mediators

An overview of the classification of mediators and their empirical support is provided in Table 1. A total of 39 potential mediators were investigated in the included RCTs. Among these, more than half of the mediators were evaluated as significant (21/39, 54%). With 48% (19/39) intervening variables, the largest group of all examined intervening variables was of a cognitive nature. A total of 13 cognitive mediators were evaluated as significant in the primary studies (13/19, 68%). We further divided the group of cognitive mediators into the *assessment* (examined: 8/39, 20%; significant: 5/8), *motivation* (examined: 1/39, 2%; significant: 0/1, 0%) and *cognitive processes* subcategories (examined: 10/39, 25%; significant: 8/10, 80%). Further evenly investigated mediator categories were emotional/affective (examined: 4/39, 10%; significant: 4/4, 100%), interpersonal (examined: 4/39, 10%; significant: 1/4, 25%), and parenting behavior mediators (examined: 4/39, 10%; significant: 0/4, 0%). The second largest group of mediators was not clearly classifiable into one of the aforementioned categories and was thus subsumed into a separate *other* mediator category (examined: 8/39, 20%; significant: 3/8, 38%). Of note, a Wilcoxon rank-sum test revealed no difference in the sample sizes between studies that found at least one significant mediator and studies that found no significant mediator (W {19,6}=59; *P*=.93).

**Table 1:** Classification of mediators.

Mediators				Studies, n (%) (n=25)		Age (years), range^a^		Guidance		Disorder		Effect size^b^		Significance		Criteria met ≥5
**Emotional and affective mediators**																
	Emotional self-perception		1 (4)		14-22		Unguided		Depression		Partially standardized ES^c^=−1.049 (95% CI −1.35 to −0.755)		Yes		(+)^d^	
	Fear		1 (4)		16-24		Internet-based psychotherapy		Depression		Between groups ES=0.49 (95% CI 0.24 to 0.75)		Yes		(+)	
	Hopelessness		1 (4)		>18		Unguided		Depression		Cohen *d*=1.13 (between groups follow-up)		Yes		(+)	
	Thought-related distress		1 (4)		Undergraduate students		Guided self-help		Generalized anxiety disorder		Cohen *d*=0.526 (between groups follow-up)		Yes		(+)	
**Interpersonal mediators**																
	Parent-Youth conflict		1 (4)		12-19		Internet-based psychotherapy		Depression		—^e^		Yes		(+)	
	Family conflicts related to diabetes management		1 (4)		12-19		Internet-based psychotherapy		Depression		—		No		(+)	
	Failed help or negative social support		1 (4)		12-19		Internet-based psychotherapy		Depression		—		No		(+)	
	Social skills		1 (4)		8-17		Guided self-help		Social phobia		—		No		(+)	
**Parenting behavior mediators**																
	Appropriate education		1 (4)		10-13		Guided self-help		Behavioral problems		—		No		(+)	
	Skill in setting clear boundaries		1 (4)		10-13		Guided self-help		Behavioral problems		—		No		(+)	
	Severity and inconsistent education		1 (4)		10-13		Guided self-help		Behavioral problems		—		No		(+)	
	Change in parenting behavior		1 (4)		3-9		Guided self-help		Behavioral problems		—		No		(−)^f^	
**Cognitive mediators**																
	**Assessment**															
		Assessment of discrepancy^g^	2^g^ (8)		College students; College students		Guided self-help; guided self-help		Risky drinking behavior		—; —		No		(+)	
		Assessment of peer drinking behavior^g^	2^g^ (8)		18-24; 18-24		Guided self-help; guided self-help		Risky drinking behavior		—; —		Yes		Different result	
		Perceived norm^g^	4^g^ (16)		18-25; Students; First semester students; 18-26		Guided self-help; unguided; guided self-help; unguided		Risky drinking behavior		—; Cohen *d*=−0.2 (ES Biannual); —; —		Different results^h^		(+)	
	**Motivation**															
		Motivation to change drinking behavior	1 (4)		College students		Unguided		Risky drinking behavior		—		No		(+)	
	**Cognitive** **processes**															
		Alcohol-related expectations	—		Students		Guided self-help		Risky drinking behavior		—		Yes		(+)	
		Remembered information	1 (4)		Students		Guided self-help		Risky drinking behavior		—		Yes		(+)	
		Mastering	1 (4)		16-25		Internet-based psychotherapy		Depression		Between groups ES=0.94 (95% CI 0.64 to 1.23)		Yes		(+)	
		Willingness to cope	—		Students		Unguided		Depression		—		No		(−)	
		Cognitive arousal before falling asleep	1 (4)		Students		Unguided		Insomnia		—		No		(+)	
		Sleep-related cognition	—		Students		Unguided		Insomnia		—		Yes		(+)	
		Postevent processing	1 (4)		8-17		Guided self-help		Social phobia		—		Yes		(+)	
		Mindful acceptance	1 (4)		Students		Guided self-help		Transdiagnostic^i^		Proportion mediated ES=range 16.05% to 28.57%		Yes		(−)	
		Obstruction of appreciation of life	—		Students		Guided self-help		Transdiagnostic^i^		Proportion mediated ES=range 29.18% to 57.94%		Yes		(−)	
		Perceived control	1 (4)		18-21		Guided self-help		Transdiagnostic^i^		Cohen *d*=0.07; Cohen *d*=0.59; Cohen *d*=0.66 (between groups follow-up)		Yes		(+)	
**Other**																
	Alcohol consumption as coping behavior		1 (4)		18-21		Unguided		Risky drinking behavior		—		No		(+)	
	Therapy adherence		1 (4)		7-18		Guided self-help		Anxiety disorder		—		No		(−)	
	Eating disorder		1 (4)		18-25		Unguided		Depression		—		Yes		(+)	
	Overall sleep quality		1 (4)		Students		Unguided		Insomnia		—		No		(+)	
	Chronotypical		1 (4)		Students		Unguided		Insomnia		—		No		(+)	
	Physical arousal before falling asleep		1 (4)		Students		Unguided		Insomnia		—		Yes		(+)	
	Trauma-related sleeping disorder		1 (4)		Students		Unguided		Insomnia		—		No		(+)	
	Insomnia		1 (4)		12-19		Guided self-help		Transdiagnostic^i^		—		Yes		(+)	

^a^If age range was not reported, participant group labels were used.

^b^Effect size measures differed across studies.

^c^ES: effect size

^d^Met 5 or more criteria.

^e^Not available.

^f^Met fewer than 5 criteria.

^g^If mediator was assessed in more than 1 study, data and results were separated with “;”.

^h^The only mediator nonsex-specific perceived norm was not significant.

^i^Transdiagnostic intervention targets more than one disorder.

A broad range of different approaches to mediation analyses was deployed, with some studies relying on several strategies at once. Bootstrapping (eg, Preacher and Hayes [71]) was used in almost half of the studies (11/25, 44%). In 32% (8/25) of the studies, the mediation analysis was based on the approach of Baron and Kenny [30], and in addition, 8% (2/25) performed a Sobel test. Furthermore, 32% (8/25) of the mediation analyses were performed using structural equation modeling (7/25, 28% using the MPlus software). Moreover, 4% (1/25) of the studies performed multiple regression analysis according to the Judd and colleagues paradigm [72], and another study did not provide any information on the statistical analysis.

### Quality Assessment of Included Studies

#### Risk-of-Bias Assessment

**Figure 2:**
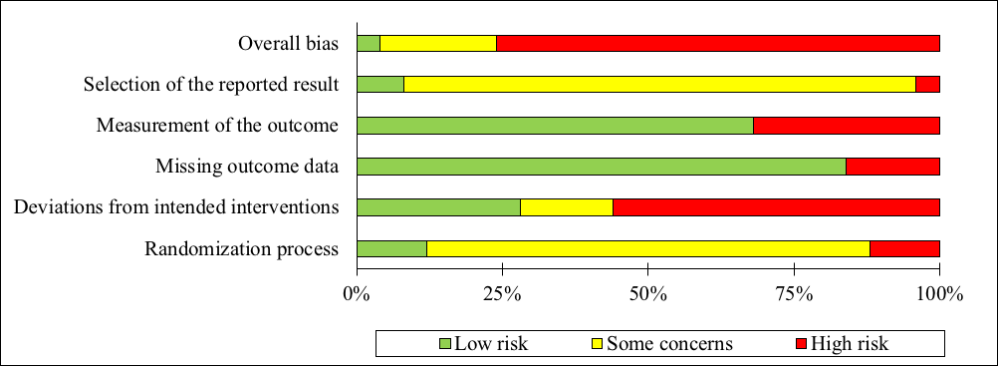
Risk-of-bias graph.

**Figure 3:**
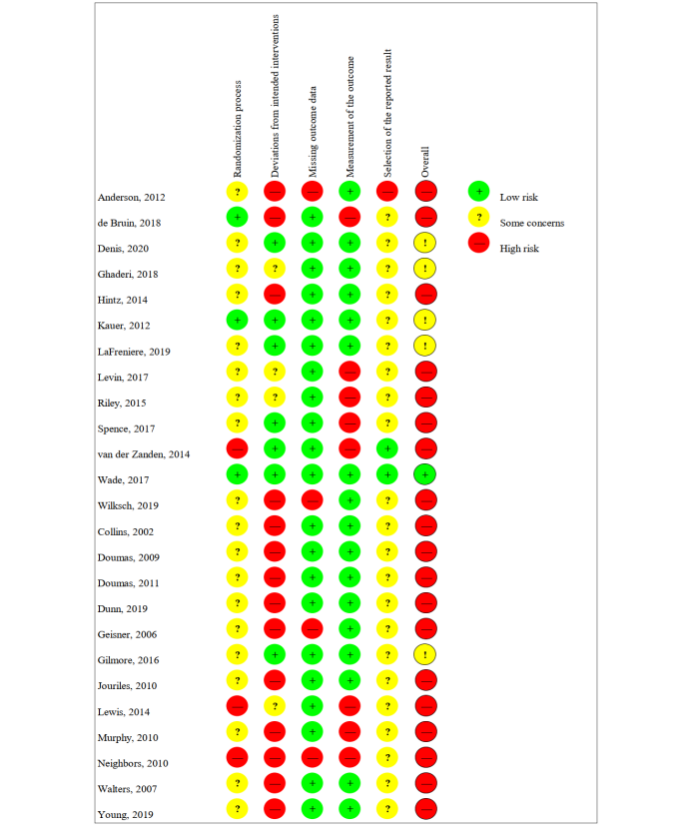
Risk-of-bias summary.

As illustrated in the *RoB2 Graph* (Figure 2), approximately three-quarters (19/25, 76%) of the included studies were rated as having a *high* RoB. In more than half of the studies (14/25, 56%), the *bias due to deviation from the intended intervention* was rated as *high*. The RoB on this domain was more often evaluated to be *high* in studies on DHI_HP_ (10/25, 40%) when compared with studies on DHI_PSY_ (4/25, 16%). Both the randomization procedure and the process of reporting results were predominantly rated with *some concern* across studies (19/25, 76% and 22/25, 88%). Interrater reliability varied across domains from Cohen κ=0.76 to Cohen κ=0.93. According to Landis and Koch [73], these agreements can be rated as *substantial* to *almost perfect*. A summary of the RoB2 assessments is shown in Figure 3.

#### Evaluation of the Quality Criteria for Process Research and Approximating Causality

The evaluation of the included studies with regard to the methodological quality of process research revealed that in both the DHI_PSY_ and the DHI_HP_ groups, most studies (DHI_PSY_: 9/13, 69% vs DHI_HP_: 11/12, 92%) fulfilled 5 or more out of the 8 criteria. Owing to the eligibility criteria used in this review, almost all studies met the requirement of an RCT design (24/25, 96%) and a CG (24/25, 96%). In the publication by Anderson et al [46], the results from 8% (2/25) of the studies were jointly reported, with only the second study evaluating a DHI_PSY_ without a direct comparison with the CG, although this criterion was fulfilled in the first study. Even if mediators were collected at more than 2 measurement time points in more than half of the studies (15/25, 60%; including follow-up), an evaluation of the chronology of changes in the mediator variable or variables and outcomes was conducted in only 4% (1/25; de Bruin et al [47]) of the studies. Furthermore, 8% (2/25) of the studies, Hintz et al [56] and Jouriles et al [57] implemented direct experimental manipulation of mediators. A detailed overview of the evaluation of the methodological quality criteria for process research and the approximation of causality are outlined in Tables 2 and 3, as well as in Multimedia Appendix 3.

**Table 2:** Quality criteria for process research and approximation of causality (n=25).

Studies		Randomized controlled trial	CG^a^	Theoretical foundation	n≥40 (Each CG and EG^b^)	Various mediators	Time sequence	Manipulation	*P* value (<.05)^c^	∑ (Yes)
**DHI_PSY_^d^**										
	Anderson, 2012 [46]^e^	No^f^	No	Yes^g^	No	No	No	No	Yes	2
	de Bruin et al [47]	Yes	Yes	Yes	No	No	Yes	No	Yes	5
	Denis et al [49]	Yes	Yes	No	Yes	Yes	No^h^	No	Yes	5
	Ghaderi et al [54]	Yes	Yes	Yes^i^	Yes	Yes	No^h^	No	Yes	6
	Hintz, 2014 [56]	Yes	Yes	Yes	Yes	No	No^h^	Yes	Yes	6
	Kauer et al [58]	Yes	Yes	Yes	Yes	No	No^h^	No	Yes	5
	LaFreniere, and Newman [59]	Yes	Yes	Yes	No	No	No^h^	No	Yes	4
	Levin et al [60]	Yes	Yes	No	No	Yes	No	No	Yes	4
	Riley et al [64]	Yes^j^	Yes^j^	Yes	Yes	Yes	No^h^	No	Yes	6
	Spence et al [65]	Yes	Yes	Yes	No	Yes	No^h,^^k^	No	Yes	5
	Zanden et al, 2014 [66]	Yes	Yes	Yes	Yes	Yes	No^h^	No	Yes	6
	Wade et al [67]	Yes	Yes	Yes	No^i^	No	No^h^	No	Yes	4
	Wilksch, O’Shea, and Wade [69]	Yes	Yes	Yes	Yes	No	No^h^	No	Yes	5
**DHI_HP_^l^**										
	Collins, Carey, and Sliwinski [48]	Yes	Yes	Yes	Yes	No	No^h^	No	Yes	5
	Doumas, McKinley, and Book [50]	Yes	Yes	Yes	No	No	No	No	Yes	4
	Doumas et al [51]	Yes	Yes	Yes	Yes	No	No	No	Yes	5
	Dunn, 2019 [52]	Yes	Yes	Yes	Yes	No	No	No	Yes	5
	Geisner, Neighbors, and Larimer [53]	Yes	Yes	Yes	Yes	Yes	No	No	Yes	6
	Gilmore and Bountress [55]	Yes	Yes	Yes	Yes	No	No	No	Yes	5
	Jouriles et al [57]	Yes	Yes	Yes	No	No	No	Yes	Yes	5
	Lewis et al [61]	Yes	Yes	Yes	Yes	No	No^h^	No	Yes	5
	Murphy, 2010 [62]^h^	Yes	Yes	Yes	Yes	Yes	No^h^	No	Yes	6
	Neighbors et al [63]	Yes	Yes	Yes	Yes	Yes	No^h^	No	Yes	6
	Walters, Vader, and Harris [68]	Yes	Yes	Yes	Yes^m^	No	No^h^	No	Yes	5
	Young and Neighbors [70]	Yes	Yes	Yes	Yes	No	No	No	Yes	5

^a^CG: control group.

^b^EG: experimental group.

^c^Overall significance level *P*<.05; only data from study 2 taken into account.

^d^DHI_PSY_: digital health interventions with a psychotherapeutic focus.

^e^Only data from study 2 are taken into account.

^f^*No* indicates criteria not met.

^g^*Yes* indicates criteria met.

^h^More than 2 measurements (including follow-up) reported, but no evaluation of time sequence.

^i^Subscales have no theoretical foundations.

^j^Initial study had a randomized controlled trial design; in secondary analysis, both groups were taken together.

^k^Due to missing follow-up data in the waitlist condition, mediation analysis was conducted only with data from baseline and after 12 weeks (at least 3 sessions were completed).

^l^DHI_HP_: digital health interventions with a focus on health promotion.

^m^Information was given after contacting authors.

**Table 3:** Number of studies meeting each single quality criterion for process research (n=25).

Criterion	DHI_PSY_^a^, n (%)	DHI_HP_^b^, n (%)	Overall, n (%)
Randomized controlled trial	12 (48)	12 (48)	24 (96)
CG^c^	12 (48)	12 (48)	24 (96)
Theoretical foundation	11 (44)	12 (48)	23 (92)
n≥40 (for CG and EG^d^ each)	7 (28)	10 (40)	17 (68)
Evaluation of various mediators	6 (24)	3 (12)	9 (36)
Time sequence or temporality	1 (4)	0 (0)	1 (4)
Manipulation of mediators	2 (8)	0 (0)	2 (8)
*P*<.05	13 (52)	12 (48)	25 (100)

^a^DHI_PSY_: digital health interventions with a psychotherapeutic focus.

^b^DHI_HP_: digital health interventions with a focus on health promotion.

^c^CG: control group.

^d^EG: experimental group.

## Discussion

### Principal Findings

This systematic review, to the knowledge of the authors the first of its kind, comprehensively evaluated research on mediators and mechanisms of change in DHIs for common mental disorders in youth. Altogether, 25 studies were included in the review, that have examined 39 distinct mediators. Cognitive variables were found to be the most often investigated mediators, followed by a broad range of other mediators. Even though our eligibility criteria were not limited to a specific mental health condition in youth, only a rather low number of studies were identified by our systematic literature searches, a finding that corresponds to the limited evidence base of research on mechanisms of change in psychotherapy in general [37,74], and for children and adolescents in specific [24,35,37], necessitating further high-quality research efforts to improve interventions and mental health care practices for this younger age group.

Remarkably, our findings indicate that the mediator category with the highest percentage of significant intervening variables was the affective or emotional mediator group (4/4, 100%). The proportions of significance in other mediator categories were by far less high and included combined cognitive (13/19, 68%), other (3/8, 37%), interpersonal (1/4, 25%), and parenting behavior–related mediators (0/4, 0%). The consistent nonsignificant findings on parenting behavior–related intervening variables are astonishing, as parenting behavior is otherwise thought to be of paramount importance in the treatment of behavioral problems in youth, both in conventional interventions delivered face-to-face [75] and in digital parent training alike [76,77]. Therefore, the findings of our study are in contrast to those of a systematic review [75], which revealed that in 45% (39/86) of the included studies on face-to-face parent training programs, parenting behavior served as a mediator for the association between the intervention and symptom change in children. Furthermore, the importance of emotion regulation might be underestimated in (digital) psychotherapeutic interventions for children and adolescents, considering the consistent pattern of significant findings across studies in our review, as well as by allowing for comprehensive earlier research highlighting the overall importance of emotion regulation competencies for mental health in childhood and adolescence [78]. However, given the rather small number of mediation studies per category in our review, these findings need to be interpreted with caution and must be considered as preliminary. This is also owing to the rather heterogeneous evidence base, where included studies varied broadly in terms of the theoretical foundations of the intervention as well as the simultaneous consideration of various mental disorders, which may further restrict the comparability between studies. Nevertheless, a Wilcoxon rank-sum test revealed no differences in the sample sizes between studies that found at least one significant mediator and studies that found no significant mediator, suggesting that there is no effect of sample size on the evaluation of intervening variables, strengthening the robustness and validity of the findings on the mediators in this regard.

Importantly, participants were most often adolescents, with only 16% (4/25) of eligible studies evaluating interventions for children, suggesting an additional research gap for this younger age group. Here, all interventions for children were conducted with the involvement of parents, suggesting a crucial role of the accompanying human support in DHIs for younger children, which must be corroborated and further specified by forthcoming research [12,79]. In studies evaluating DHI_HP_, only cognitive mediators (perceived norm and alcohol-related expectation) were investigated. This finding is consistent with prior research, where the cognitive mediator, perceived norm, was established as one of the most evaluated mediators in interventions for problematic drinking behaviors in adolescents and young adults delivered on site [80]. However, this result rather highlights the researcher’s presumptions than the evidence base of the superior relevance of cognitive mediators over other, not yet well examined, affective and behavioral mediators.

The results of the RoB assessment indicated a rather limited overall study quality, with 76% (19/25) of the included studies rated with a *high RoB,* a finding that aligns with the review by Christ et al [81], in which 92% (22/24) included studies on DHIs for adolescents and young adults were assessed with a *high RoB*. These findings on RoB2 might be largely due to the specifics of psychotherapy research [82], where masking of therapists or personnel and participants is difficult to achieve, as well as the more conservative novel RoB2 algorithm [83]. Of note, in our review, the mean study dropout rate was 18%. Therefore, only a fraction of the included studies fulfilled the RoB2 criterion that 95% of randomized participants’ data should be available for data analysis. Although the current mean dropout rate in this review is rather small compared with other high dropout rates found in DHIs, the well-known issue of limited engagement still warrants future research to further investigate approaches to remedy (study and intervention) attrition [84,85]. In this particular field, primary studies in the review at hand may offer guidance and direction to more effective engagement in youth (Multimedia Appendix 2), such as interactive intervention components and age-appropriate content presentation in the form of puzzles, videos, or cartoons [12].

The results of the methodological quality assessment for process research revealed that most studies adhered to certain quality criteria satisfactorily (ie, using an RCT design and CG, evaluation of a strong statistical association, describing a theoretical foundation for mediators and sample size per trial group). However, in contrast, most primary studies did not experimentally manipulate the potential mediators, did not assess several mediators simultaneously, and did not evaluate the time sequence or temporal ordering of changes in mediators and outcomes. However, this latter criterion is considered to be of utmost importance for causal inferences and is sometimes referred to as the *fifth step* of mediation analysis [86]. Even though mediators and outcomes were assessed more than twice in 60% (15/25) of the included studies, only 4% (1/25) of the studies [47] actually conducted a statistical evaluation of the time sequence of cause and effect. Taken together, the findings of our review seem to be in line with those of other research [24,44,45,83], pointing to important methodological shortcomings of mediation studies, which should be amended by future research. At this point, 1 high-quality RCT [58] sets an example, fulfilling 6 out of 8 quality criteria.

### Strengths and Limitations

This systematic review offers several strengths, including a comprehensive literature search, as well as the broad consideration of common mental disorders and theoretical orientations, resulting in an extensive overview of the research on mediators and mechanisms of change for DHIs in children and adolescents conducted so far. Furthermore, with the differential consideration of the 2 categories of DHI_PSY_ and DHI_HP_, we intended to provide specific evidence-based information that might be valuable for digital psychotherapeutic and health promotion interventions alike. However, some limitations must be considered when interpreting the findings of this review. First, the number of eligible studies is rather small; the generalizability of the findings might be reduced as most studies were conducted in Western countries; these studies evaluated internet-based interventions; participants were predominantly female; and these studies relied on older participants. Second, only English language papers were included, and we did not incorporate gray literature in our study as recent findings indicated a negligible relevance of gray literature in systematic reviews [87]; further, publication bias cannot be ruled out. Third, although we only included RCT studies in our review (which might have led to an overestimation of the study quality in general, omitting other design studies with potentially lower methodological rigor), the RoB assessment indicated substantial shortcomings in the included studies. However, this finding should be weighed against the typical constraints of research on psychological interventions.

### Future Directions

The following recommendations might be helpful for future mediation studies, aiming to advance our understanding of the working mechanisms of DHIs for youth. First, it is essential to avoid the methodological shortcomings outlined in the assessment of quality criteria for process research, especially with regard to the temporality of changes and experimental manipulation of mediators, both of which are key for the justification of causal inferences. Furthermore, future studies should resort to more sophisticated and current methods of mediation analysis (eg, Grimm et al [88] and Hofmann et al [89]), ideally capable of clarifying temporal precedence and illustrating the actual pattern of change [24]. Second, the therapeutic or working alliance (ie, the professional relationship between therapists and patients; eg, Grawe et al [90]) was not evaluated as a mediator in any of the included studies. However, the recent development of the working alliance inventory for digital interventions [91] may contribute to future process research, informing the evidence base of the effects of a *digital* therapeutic alliance in DHIs for youth [37,92]. Furthermore, mediators that have not been investigated so far, such as behavioral and biological variables, should be evaluated in future studies. At this point, the possibilities of novel technological methods in process research seem not to be fully exploited in this research field [37]. Passive sensing methodologies and digital phenotyping approaches with smartphones [93] might generate insights on behavioral and biological mechanisms in real life, in addition to the predominant information on cognitive variables derived from self-reports identified in the current review. Third, although worldwide about 90% of youth live in countries with low or medium income, 90% of RCTs investigating mental disorders in children and adolescents are conducted in high-income countries [1], mirroring the findings of the review at hand. In contrast, the presumed advantages of DHIs may be especially relevant in structurally weak and low-income countries. To overcome this contradiction, replication studies should aim to include and reach populations from low-income countries as well. Finally, our review revealed that mediators focusing on emotional and cognitive processes might be of paramount importance as potential mechanisms of change in DHIs for youth. However, the processes covered by these mediators varied to some extent. Thus, future studies focusing on both emotional and cognitive processes in a systematic way within one framework could be of great importance for the field.

### Conclusions

This systematic review is the first to comprehensively investigate the mechanisms of change in different DHIs for youth. The key findings indicate that the largest group of examined mediators are cognitive variables, followed by an array of other mediator variables, including interpersonal, parenting behavior, and affective mediator categories. Of note, emotional and affective mediators consistently reached statistical significance across studies, whereas parenting-related mediators were evaluated constantly as nonsignificant. However, these findings must be considered cautiously, as we detected only a limited number of primary studies, despite including a broad range of mental disorders and interventions. Future studies should aim to lessen this research gap, ideally adhering to the quality criteria for process research and recent methods of mediation analyses, enabling more causally robust findings. These forthcoming studies might contribute to disentangling the therapeutic processes in DHIs, providing evidence-based knowledge to inform intervention development and augmented mental health care practices worldwide.

## Appendix

Multimedia Appendix 1 Search strings for Cochrane Central Register of Controlled Trials, Embase, MEDLINE, and PsycINFO in Ovid.

Multimedia Appendix 2 Study characteristics.

Multimedia Appendix 3 Studies with number of criteria met.

## Abbreviations

CG: control group
DHI: digital health intervention
DHIHP: digital health intervention with a focus on health promotion
DHIPSY: digital health intervention with a psychotherapeutic focus
RCT: randomized controlled trial
RoB: risk of bias
