# Supplementary material for: Mechanisms of Change in Digital Health Interventions for Mental Disorders in Youth: Systematic Review
Source: J Med Internet Res. 2021 Nov 26;23(11):e29742. doi: 10.2196/29742 (PMC8665396; doi:10.2196/29742)
Supplement: Multimedia Appendix 1 [file jmir_v23i11e29742_app1.docx]

**Multimedia Appendix 1.**

Mechanisms of Change in Digital Health Interventions for Mental Disorders in Youth: Systematic Review

| *Multimedia Appendix 1. Table S.1 Search strings for CENTRAL, Embase, MEDLINE and PsycINFO in Ovid* |
| --- |

| No. | CENTRAL | Embase | MEDLINE^a^ | PsycINFO |  |
| --- | --- | --- | --- | --- | --- |
|  | *exp child/^b^* | *exp child/* | *exp child/* | *exp childhood development/* |  |
|  | *exp adolescent/* | *exp adolescent/* | *exp adolescent/* | *exp adolescent development/* |  |
|  | *exp infant/* | *exp infant/* | *exp infant/* | *exp infant development/* |  |
|  | *exp puberty/* | *exp puberty/* | *exp puberty/* | *exp puberty/* |  |
|  | boy*.ti,ab,kw. | boy*.ti,ab,kw. | boy*.ti,ab,kw. | boy*.ti,ab,id. |  |
|  | girl*.ti,ab,kw. | girl*.ti,ab,kw. | girl*.ti,ab,kw. | girl*.ti,ab,id. |  |
|  | male*.ti,ab,kw. | male*.ti,ab,kw. | male*.ti,ab,kw. | male*.ti,ab,id. |  |
|  | female*.ti,ab,kw. | female*.ti,ab,kw. | female*.ti,ab,kw. | female*.ti,ab,id. |  |
|  | juvenil*.ti,ab,kw. | juvenil*.ti,ab,kw. | juvenil*.ti,ab,kw. | juvenil*.ti,ab,id. |  |
|  | minor*.ti,ab,kw. | minor*.ti,ab,kw. | minor*.ti,ab,kw. | minor*.ti,ab,id. |  |
|  | p?ediatric*.ti,ab,kw. | p?ediatric*.ti,ab,kw. | p?ediatric*.ti,ab,kw. | p?ediatric*.ti,ab,id. |  |
|  | kindergarten*.ti,ab,kw. | kindergarten*.ti,ab,kw. | kindergarten*.ti,ab,kw. | kindergarten*.ti,ab,id. |  |
|  | nurser*.ti,ab,kw. | nurser*.ti,ab,kw. | nurser*.ti,ab,kw. | nurser*.ti,ab,id. |  |
|  | *exp child,preschool/* | *exp preschool child/* | *exp child,preschool/* | *exp preschool students/* |  |
|  | school*.ti,ab,kw. | school*.ti,ab,kw. | school*.ti,ab,kw. | school*.ti,ab,id. |  |
|  | pre adolesc*.ti,ab,kw. | pre adolesc*.ti,ab,kw. | pre adolesc*.ti,ab,kw. | pre adolesc*.ti,ab,id. |  |
|  | pre-adolesc*.ti,ab,kw. | pre-adolesc*.ti,ab,kw. | pre-adolesc*.ti,ab,kw. | pre-adolesc*.ti,ab,id. |  |
|  | pre pube*.ti,ab,kw. | pre pube*.ti,ab,kw. | pre pube*.ti,ab,kw. | pre-pube*.ti,ab,id. |  |
|  | pre-pube*.ti,ab,kw. | pre-pube*.ti,ab,kw. | pre-pube*.ti,ab,kw. | pre pube*.ti,ab,id. |  |
|  | high school*.ti,ab,kw. | high school*.ti,ab,kw. | high school*.ti,ab,kw. | high school*.ti,ab,id. |  |
|  | high-school*.ti,ab,kw. | high-school*.ti,ab,kw. | high-school*.ti,ab,kw. | high-school*.ti,ab,id. |  |
|  | youth*.ti,ab,kw. | youth*.ti,ab,kw. | youth*.ti,ab,kw. | youth*.ti,ab,id. |  |
|  | teen*.ti,ab,kw. | teen*.ti,ab,kw. | teen*.ti,ab,kw. | teen*.ti,ab,id. |  |
|  | student*.ti,ab,kw. | student*.ti,ab,kw. | student*.ti,ab,kw. | student*.ti,ab,id. |  |
|  | undergrad*.ti,ab,kw. | undergrad*.ti,ab,kw. | undergrad*.ti,ab,kw. | undergrad*.ti,ab,id. |  |
|  | college*.ti,ab,kw. | college*.ti,ab,kw. | college*.ti,ab,kw. | college*.ti,ab,id. |  |
|  | universit*.ti,ab,kw. | universit*.ti,ab,kw. | universit*.ti,ab,kw. | universit*.ti,ab,id. |  |
|  | 1 or 2 or 3 or 4 or 5 or 6 or 7 or 8 or 9 or 10 or 11 or 12 or 13 or 14 or 15 or 16 or 17 or 18 or 19 or 20 or 21 or 22 or 23 or 24 or 25 or 26 or 27 | 1 or 2 or 3 or 4 or 5 or 6 or 7 or 8 or 9 or 10 or 11 or 12 or 13 or 14 or 15 or 16 or 17 or 18 or 19 or 20 or 21 or 22 or 23 or 24 or 25 or 26 or 27 | 1 or 2 or 3 or 4 or 5 or 6 or 7 or 8 or 9 or 10 or 11 or 12 or 13 or 14 or 15 or 16 or 17 or 18 or 19 or 20 or 21 or 22 or 23 or 24 or 25 or 26 or 27 | 1 or 2 or 3 or 4 or 5 or 6 or 7 or 8 or 9 or 10 or 11 or 12 or 13 or 14 or 15 or 16 or 17 or 18 or 19 or 20 or 21 or 22 or 23 or 24 or 25 or 26 or 27 |  |
|  | *exp mental disorders/* | *exp mental disorders/* | *exp mental disorders/* | *exp mental disorders/* |  |
|  | *exp mental health/* | *exp mental health/* | *exp mental health/* | *exp mental health/* |  |
|  | *exp child psychiatry/* | *exp child psychiatry/* | *exp child psychiatry/* | *exp child psychiatry/* |  |
|  | *exp adolescent psychiatry/* | *-* | *exp adolescent psychiatry/* | *exp adolescent psychiatry/* |  |
|  | mental ill*.ti,ab,kw. | mental ill*.ti,ab,kw. | mental ill*.ti,ab,kw. | mental ill*.ti,ab,id. |  |
|  | mental distress.ti,ab,kw. | mental distress.ti,ab,kw. | mental distress.ti,ab,kw. | mental distress.ti,ab,id. |  |
|  | 29 or 30 or 31 or 32 or 33 or 34 | 29 or 30 or 31 or 33 or 34 | 29 or 30 or 31 or 32 or 33 or 34 | 29 or 30 or 31 or 32 or 33 or 34 |  |
|  | *exp internet/* | *exp internet/* | *exp internet/* | *exp internet/* |  |
|  | online*.ti,ab,kw. | online*.ti,ab,kw. | online*.ti,ab,kw. | online*.ti,ab,id. |  |
|  | web*.ti,ab,kw. | web*.ti,ab,kw. | web*.ti,ab,kw. | web*.ti,ab,id. |  |
|  | digital*.ti,ab,kw. | digital*.ti,ab,kw. | digital*.ti,ab,kw. | digital*.ti,ab,id. |  |
|  | virtual*.ti,ab,kw. | virtual*.ti,ab,kw. | virtual*.ti,ab,kw. | virtual*.ti,ab,id. |  |
|  | computer*.ti,ab,kw. | computer*.ti,ab,kw. | computer*.ti,ab,kw. | computer*.ti,ab,id. |  |
|  | mobile*.ti,ab,kw. | mobile*.ti,ab,kw. | mobile*.ti,ab,kw. | mobile*.ti,ab,id. |  |
|  | smartphone*.ti,ab,kw. | smartphone*.ti,ab,kw. | smartphone*.ti,ab,kw. | smartphone*.ti,ab,id. |  |
|  | e mail*.ti,ab,kw. | e mail*.ti,ab,kw. | e mail*.ti,ab,kw. | e mail*.it,ab,id. |  |
|  | e-mail*.ti,ab,kw. | e-mail*.ti,ab,kw. | e-mail*.ti,ab,kw. | e-mail*.ti,ab,id. |  |
|  | app*.ti,ab,kw. | app*.ti,ab,kw. | app*.ti,ab,kw. | app*.ti,ab,id. |  |
|  | cyber*.ti,ab,kw. | cyber*.ti,ab,kw. | cyber*.ti,ab,kw. | cyber*.ti,ab,id. |  |
|  | phone*.ti,ab,kw. | phone*.ti,ab,kw. | phone*.ti,ab,kw. | phone*.ti,ab,id. |  |
|  | avatar*.ti,ab,kw. | avatar*.ti,ab,kw. | avatar*.ti,ab,kw. | avatar*.ti,ab,id. |  |
|  | wireless technolog*.ti,ab,kw. | wireless technolog*.ti,ab,kw. | wireless technolog*.ti,ab,kw. | wireless technolog*.ti,ab,id. |  |
|  | virtual realit*.ti,ab,kw. | virtual realit*.ti,ab,kw. | virtual realit*.ti,ab,kw. | virtual realit*.ti,ab,id. |  |
|  | e health*.ti,ab,kw. | e health*.ti,ab,kw. | e health*.ti,ab,kw. | e health*.ti,ab,id. |  |
|  | e-health*.ti,ab,kw. | e-health*.ti,ab,kw. | e-health*.ti,ab,kw. | e-health*.ti,ab,id. |  |
|  | chat*.ti,ab,kw. | chat*.ti,ab,kw. | chat*.ti,ab,kw. | chat*.ti,ab,id. |  |
|  | videoconferenc*.ti,ab,kw. | videoconferenc*.ti,ab,kw. | videoconferenc*.ti,ab,kw. | videoconferenc*.ti,ab,id. |  |
|  | 36 or 37 or 38 or 39 or 40 or 41 or 42 or 43 or 44 or 45 or 46 or 47 or 48 or 49 or 50 or 51 or 52 or 53 or 54 or 55 | 36 or 37 or 38 or 39 or 40 or 41 or 42 or 43 or 44 or 45 or 46 or 47 or 48 or 49 or 50 or 51 or 52 or 53 or 54 or 55 | 36 or 37 or 38 or 39 or 40 or 41 or 42 or 43 or 44 or 45 or 46 or 47 or 48 or 49 or 50 or 51 or 52 or 53 or 54 or 55 | 36 or 37 or 38 or 39 or 40 or 41 or 42 or 43 or 44 or 45 or 46 or 47 or 48 or 49 or 50 or 51 or 52 or 53 or 54 or 55 |  |
|  | *exp psychotherapy/* | *exp psychotherapy/* | *exp psychotherapy/* | *exp psychotherapy/* |  |
|  | *-* | *-* | *-* | *exp child psychotherapy/* |  |
|  | *-* | *-* | *-* | *exp adolescent psychotherapy/* |  |
|  | *exp counseling/* | *exp counseling/* | *exp counseling/* | *exp counseling/* |  |
|  | therap*.ti,ab,kw. | therap*.ti,ab,kw. | therap*.ti,ab,kw. | therap*.ti,ab,id. |  |
|  | treatment*.ti,ab,kw. | treatment*.ti,ab,kw. | treatment*.ti,ab,kw. | treatment*.ti,ab,id. |  |
|  | intervention*.ti,ab,kw. | intervention*.ti,ab,kw. | intervention*.ti,ab,kw. | intervention*.ti,ab,id. |  |
|  | self-help*.ti,ab,kw. | self-help*.ti,ab,kw. | self-help*.ti,ab,kw. | self-help*.ti,ab,id. |  |
|  | training*.ti,ab,kw. | training*.ti,ab,kw. | training*.ti,ab,kw. | training*.ti,ab,id. |  |
|  | 57 or 60 or 61 or 62 or 63 or 64 or 65 | 57 or 60 or 61 or 62 or 63 or 64 or 65 | 57 or 60 or 61 or 62 or 63 or 64 or 65 | 57 or 58 or 59 or 60 or 61 or 62 or 63 or 64 or 65 |  |
|  | *exp telemedicine/* | *exp telemedicine/* | *exp telemedicine/* | *exp telemedicine/* |  |
|  | *exp therapy, computer-assisted/* | *exp computer assisted therapy/* | *exp therapy, computer-assisted/* | *exp computer assisted therapy/* |  |
|  | *-* | *-* | *-* | *exp online therapy/* |  |
|  | e counsel*.ti,ab,kw. | e counsel*.ti,ab,kw. | e counsel*.ti,ab,kw. | e counsel*.ti,ab,id. |  |
|  | e-counsel*.ti,ab,kw. | e-counsel*.ti,ab,kw. | e-counsel*.ti,ab,kw. | e-counsel*.ti,ab,id. |  |
|  | e therap*.ti,ab,kw. | e therap*.ti,ab,kw. | e therap*.ti,ab,kw. | e therap*.ti,ab,id. |  |
|  | e-therap*.ti,ab,kw. | e-therap*.ti,ab,kw. | e-therap*.ti,ab,kw. | e-therap*.ti,ab,id. |  |
|  | distance counsel*.ti,ab,kw. | distance counsel*.ti,ab,kw. | distance counsel*.ti,ab,kw. | distance counsel*.ti,ab,id. |  |
|  | (online adj2 therap*).ti,ab,kw. | (online adj2 therap*).ti,ab,kw. | (online adj2 therap*).ti,ab,kw. | (online adj2 therap*).ti,ab,id. |  |
|  | ecological momentary intervention*.ti,ab,kw. | ecological momentary intervention*.ti,ab,kw. | ecological momentary intervention*.ti,ab,kw. | ecological momentary intervention*.ti,ab,id. |  |
|  | 67 or 68 or 70 or 71 or 72 or 73 or 74 or 75 or 76 | 67 or 68 or 70 or 71 or 72 or 73 or 74 or 75 or 76 | 67 or 68 or 70 or 71 or 72 or 73 or 74 or 75 or 76 | 67 or 68 or 69 or 70 or 71 or 72 or 73 or 74 or 75 or 76 |  |
|  | - | *exp controlled clinical trial/* | *exp controlled clinical trial/* | *exp clinical trials/* |  |
|  | - | radom*.ti,ab,kw. | radom*.ti,ab,kw. | random*.ti,ab,id. |  |
|  | - | (random* adj3 controlled).ti,ab,kw. | (random* adj3 controlled).ti,ab,kw. | (random* adj3 controlled).ti,ab,id. |  |
|  | - | RCT*.ti,ab,kw. | RCT*.ti,ab,kw. | RCT*.ti,ab,id. |  |
|  | - | 78 or 79 or 80 or 81 | 78 or 79 or 80 or 81 | 78 or 79 or 80 or 81 |  |
|  | mediat*.ti,ab,kw. | mediat*.ti,ab,kw. | mediat*.ti,ab,kw. | mediat*.ti,ab,id. |  |
|  | mechanism* of change.ti,ab,kw. | mechanism* of change.ti,ab,kw. | mechanism* of change.ti,ab,kw. | mechanism* of change.ti,ab,id. |  |
|  | mechanism* of psychotherapy.ti,ab,kw. | mechanism* of psychotherapy.ti,ab,kw. | mechanism* of psychotherapy.ti,ab,kw. | mechanism* of psychotherapy.ti,ab,id. |  |
|  | change mechanism*.ti,ab,kw. | change mechanism*.ti,ab,kw. | change mechanism*.ti,ab,kw. | change mechanism*.ti,ab,id. |  |
|  | process$2.it,ab,kw. | process$2.ti,ab,kw. | process$2.ti,ab,kw. | process$2.it,ab,id. |  |
|  | therap* change.ti,ab,kw. | therap* change.ti,ab,kw. | therap* change.ti,ab,kw. | therap* change.ti,ab,id. |  |
|  | mediation analys*.ti,ab,kw. | mediation analys*.ti,ab,kw. | mediation analys*.ti,ab,kw. | mediation analys*.ti,ab,id. |  |
|  | indirect effect*.ti,ab,kw. | indirect effect*.ti,ab,kw. | indirect effect*.ti,ab,kw. | indirect effect*.ti,ab,id. |  |
|  | working function*.ti,ab,kw. | working function*.ti,ab,kw. | working function*.ti,ab,kw. | working function*.ti,ab,id. |  |
|  | intervention variable*.ti,ab,kw. | intervention variable*.ti,ab,kw. | intervention variable*.ti,ab,kw. | intervention variable*.ti,ab,id. |  |
|  | third variable*.ti,ab,kw. | third variable*.ti,ab,kw. | third variable*.ti,ab,kw. | third variable*.ti,ab,id. |  |
|  | working mechanism*.ti,ab,kw. | working mechanism*.ti,ab,kw. | working mechanism*.ti,ab,kw. | working mechanism*.ti,ab,id. |  |
|  | active  ingredient*.ti,ab,kw. | active  ingredient*.ti,ab,kw. | active ingredient*.ti,ab,kw. | active ingredient*.ti,ab,id. |  |
|  | therapeutic agent*.ti,ab,kw. | therapeutic agent*.ti,ab,kw. | therapeutic agent*.ti,ab,kw. | therapeutic agent*.ti,ab,id. |  |
|  | specific factor*.ti,ab,kw. | specific factor*.ti,ab,kw. | specific factor*.ti,ab,kw. | specific factor*.ti,ab,id. |  |
|  | common factor*.ti,ab,kw. | common factor*.ti,ab,kw. | common factor*.ti,ab,kw. | common factors*.ti,ab,id |  |
|  | working alliance*.ti,ab,kw. | working alliance*.ti,ab,kw. | working alliance*.ti,ab,kw. | working alliance*.ti,ab,id. |  |
|  | therapeutic alliance*.ti,ab,kw. | therapeutic alliance*.ti,ab,kw. | therapeutic alliance*.ti,ab,kw. | therapeutic alliance*.ti,ab,id. |  |
|  | expectation*.ti,ab,kw. | expectation*.ti,ab,kw. | expectation*.ti,ab,kw. | expectation*.ti,ab,id. |  |
|  | cognitive factor*.ti,ab,kw. | cognitive factor*.ti,ab,kw. | cognitive factor*.ti,ab,kw. | cognitive factor*.ti,ab,id. |  |
|  | behavio?ral factor*.ti,ab,kw. | behavio?ral factor*.ti,ab,kw. | behavio?ral factor*.ti,ab,kw. | behavio?ral factor*.ti,ab,id. |  |
|  | emotional factor*.ti,ab,kw. | emotional factor*.ti,ab,kw. | emotional factor*.ti,ab,kw. | emotional factor*.ti,ab,id. |  |
|  | affective factor*.ti,ab,kw. | affective factor*.ti,ab,kw. | affective factor*.ti,ab,kw. | affective factor*.ti,ab,id. |  |
|  | 83 or 84 or 85 or 86 or 87 or 88 or 89 or 90 or 91 or 92 or 93 or 94 or 95 or 96 or 97 or 98 or 99 or 100 or 101 or 102 or 103 or 104 or 105 | 83 or 84 or 85 or 86 or 87 or 88 or 89 or 90 or 91 or 92 or 93 or 94 or 95 or 96 or 97 or 98 or 99 or 100 or 101 or 102 or 103 or 104 or 105 | 83 or 84 or 85 or 86 or 87 or 88 or 89 or 90 or 91 or 92 or 93 or 94 or 95 or 96 or 97 or 98 or 99 or 100 or 101 or 102 or 103 or 104 or 105 | 83 or 84 or 85 or 86 or 87 or 88 or 89 or 90 or 91 or 92 or 93 or 94 or 95 or 96 or 97 or 98 or 99 or 100 or 101 or 102 or 103 or 104 or 105 |  |
|  | 56 and 66 | 56 and 66 | 56 and 66 | 56 and 66 |  |
|  | 77 or 107 | 77 or 107 | 77 or 107 | 77 or 107 |  |
|  | 28 and 35 and 106 and 108 | 28 and 35 and 82 and 106 and 108 | 28 and 35 and 82 and 106 and 108 | 28 and 35 and 82 and 106 and 108 |  |
| *Note*. $<n> = limits the characters that follow the given word to a certain number (n), *<term> = various versions of a word with different suffixes, ? = indicates zero or one characters within or at the end of a word, ab = abstract, adj(n) = position operator that retrieves the requested terms within a certain number (n) of words in any order, exp = explode (provide an enlarged search for the requested terms and include more related terms), id = key concepts, kw = keyword*,* ti = title.  ^a^ = Medline databases are searched: Ovid MEDLINE®, Ovid MEDLINE® Epub Ahead of Print, Ovid MEDLINE®In-Process & Other Non-Indexed Citations, Ovid MEDLINE® Daily Update.  ^b^ = Thesaurus terms are written in italics. | | | | | |
