# Supplementary material for: Mechanisms of Change in Digital Health Interventions for Mental Disorders in Youth: Systematic Review
Source: J Med Internet Res. 2021 Nov 26;23(11):e29742. doi: 10.2196/29742 (PMC8665396; doi:10.2196/29742)
Supplement: Multimedia Appendix 2 [file jmir_v23i11e29742_app2.docx]

**Multimedia Appendix 2.**

Mechanisms of Change in Digital Health Interventions for Mental Disorders in Youth: Systematic Review

*Multimedia Appendix 2. Table S.2 Study characteristics*

| Study  (Country) | Sample | Mean age (*SD*) / Age range (years) /percentage female (%) | Mental disorder^a^ / measurement instrument of symptom change (MTP in weeks^b^) | Interventions (*n*) | Degree of support^c^ | Modules (number) / duration (in weeks) | Study drop-out rate (%)^d^ | Completed modules (*SD*) / duration of use / Participants who completed all modules | Potential mediators (* / n.s.) | Measurement instrument of mediators (MTP in weeks) | Statistical method of mediation analysis (authors and year) |
| --- | --- | --- | --- | --- | --- | --- | --- | --- | --- | --- | --- |
| DHI_PSY_ | | |  |  |  |  |  |  |  |  |  |
| Anderson,  2012  (AUS)^e^  [46] | *N* = 132  Children/adolescents (ADIS-C/P ≥ 4) and at least one parent | 12.12 (2.50)  / 7 – 18 /  53.03 | Anxiety disorder /  CGAS  (0 / - / 26.07) | iCBT (*BRAVE*)  (132)  vs. ‐ (‐) | (ii) | Children/adolescents:  10 + 2;  Parents of children  6 + 2;  Parents of adolescents 5 + 2 / 12 | ‐ | Children/Adolescents: 8.86 (1.90);  Parents of children: 5.74 (0.66); Parents of adolescents: 4.76 (0.56)  / - /  Children/adolescents: 85%; Parents: 89% | ↑ adherence^f^ (n.s.) | Percentage of modules and homework completed  (- / -/ 26.07) | According to Baron and Kenny (1986); Bootstrapping (Hayes et al., 2009) |
| de Bruin,  2018  (NLD)  [47] | *N* = 116  Adolescents with insomnia according to DSM-V criteria | 15.6 (1.6)  / 12–19 /  75 | Transdiagnostic Transdiagnostisch / YSR  (0 / 6 / 8.7, 26.07, 104.29, 208.57)^g^ | CBT with focus on insomnia:  via Internet (39)  vs.  via F2F (38)  vs.  WL (39) | (ii) | 6 + 1 / 6 | 1% | ‐ / - / - | ↓ insomnia symptoms (*affective disorder, anxiety disorder, partial ADHD) | HSDQi  (0 / 6 / 8.7, 26.07, 104.29, 208.57^h^) | Multilevel regression analysis; Bootstrapping (Hayes, 2013;  MacKinnon et al., 2002; Preacher & Hayes, 2004) |
| Denis,  2020^i^  (GBR)  [49] | *N* = 199  female students | 20 (5)  / - /  100 | Insomnia  SCI  (0 / 3, 6 / 26.07) | iCBT with focus on insomnia (99) (*Sleepio*)  vs.  Online-Puzzle (100) | (i) | 1. / 6 | 27% | ‐/ - / 70.07 %^j^ | ↑ general sleep quality (n.s.);  ↑ sleep-related cognitions (*);  ↓ somatic arousal before falling asleep (*);  ↓ cognitive arousal before falling asleep (n.s.);  ↓ trauma-related sleeping disorders (n.s.);  ↓ Chronotype (n.s.) | PSQI  (0 / 3, 6 / 26.07^k^);  DBAS  (0 / 3, 6 / 26.07^k^);  PSAS  (0 / 3, 6 / 26.07^k^);  PSAS  (0 / 3, 6 / 26.07^k^);  PSQI-A  (0 / 3, 6 / 26.07^k^);  MCTQ  (0 / 3, 6 / 26.07^k^) | Bootstrapped mediated regression model (authors not specified) |
| Ghaderi,  2018  (SWE)  [54] | *N* = 231 families with a child  (SDQ ≥ 3) | ‐  / 10 – 13 /  - | Behavioral problems /  DBD, SDQ  (0 / 10 / 52.14, 104.28) | Parent training (122) *(FCU)*  vs.  internet-based parent training  (109)  *(iComet)* | (ii) | ‐ / - ;  7 / 10 | 39% | ‐ / - / -  7.7 (5.0) of 15 tasks  / - /- | ↓ strict and inconsistent education (n.s.);  ↑ appropriate education (n.s.);  ↑ set boundaries (n.s.) | PPI (0/ 5, 10/ -) | PROCESS  (Hayes, 2012) |
| Hintz,  2015  (USA)  [56] | *N* = 292  students (PCOSES ≤ 3) | ‐  / 18 – 21 (77 %) /  70 | Transdiagnostic / DASS-21  (0 / 1-2 / 3) | Online stress management intervention: PCI (97)  vs.  PCI plus fFeedback (98)  vs.  information about stress (97) | (ii) | 4 / 1 – 2^l^ | 30% | ‐ / - / - | ↑ perceived control (*) | PCOSES  (0 / 1 – 2^l^ / 3) | SPSS macro for multiple mediation analysis  (Preacher & und Hayes, 2008) |
| Kauer,  2012  (AUS)  [58] | *N* = 118  adolescents with mild/severe emotional/mental problems (assessed by the general practitioner or K10 > 16) | 18.06 (3.2)^n^  / 14 – 24 /  71.92^p^ | depression/  DASS  (0 / 2 – 4 / 6) | self-monitoring (69)  vs. attention control group (49) | (i) | 8 / 2 – 4 | 26% | 3.3 (1.42)  Entries per day  / - / On 17.7 (6.69) days the complete programme was executed | ↑ emotional self-perception (*) | ESA-Scale  (0 / 2 – 4 / 6) | Structural equation model;  Bootstrapping  (Cheong et al., 2003; Taylor et al., 2008) in Mplus 6 (Muthén & Muthén, 1998 – 2011) |
| LaFreniere,  2019^o^  (USA)  [59] | *N* = 51 students with generalized anxiety disorder  (GAD-Q-IV) | 18.82 (1.07)^p^  / - /  84.31 | Generalized anxiety disorder /  PSWQ  (0/ 1.57 / 4.29) | Ecological Momentary Intervention:  WOJ (29)  vs.  TL (22) | (ii) | 4 per day/ 1.14 | 2% | ‐ / - / - | ↓ thought-related distress (*)^q^ | MCQ  (0 / 1.57 / 4.29) | Bootstrapping,  regression-based path analysis  (Preacher & Hayes, 2008;  Hayes, 2009)  in Mplus 7 (Muthén & Muthén, 2012) |
| Levin,  2017  (USA)  [60] | *N* = 79 students | 20.51 (2.73)  / - /  66 | Transdiagnostic / CCAPS-34  (0 / 4 / -) | Web-based ACT (40)  vs.  WL(39) | (ii) | 6 / 4 | 21% | - / 78.58 Min. (83.28) /  55 % | ↑ mindfulness (*)^r^  ↓ obstruction of the appreciation of life (*)^r^ | PHLMS  (0 / 4 / -)  VQ  (0 / 4 / -) | Cross product of the coefficient test with bootstrapping in Mplus 7 (Muthén & Muthén, 2012) |
| Riley,  2015^s^  (USA)  [64] | *N* = 90  adolescents with diabetes type-1 and at least one parent | 14.8 (1.6)^t^  / 12 – 19 /  41.5 | depression/  CDI  (0 / 12 / 13.04) | BFST-D  via Skype (46)^u;v^  vs.  BFST-D via F2F (44)^u;v^ | (iii) | 10 / 12 | 12% | 6.3 (3.4)  / - / 23.2 % | ↓ parent-youth conflict (*)  ↓ diabetes family conflict (n.s.)  ↓ failure of assistence (n.s.) | CBQ  (0 / 12 / 13.04)  DFCS  (0 / 12 / 13.04)  HHI  (0 / 12 /13.04) | Multiple regression analysis  (Judd et al., 2001) |
| Spence,  2017  (AUS)  [65] | *N* = 125  Children/adolescents/  (ADIS-C/P ≥ 4) and at least one parent | 11.29 (2.67)^t^  / 8 – 17 /  60 | Social phobia/ SPAI-C/P,  ADIS-C/P  (0 / 12 / 26.07) | iCBT social phobia (47)^u^  vs.  iCBT conventional  (*BRAVE*) (48)^u^  vs.  WL (30) | (ii) | Children/adolescents  10 + 2;  Parents of children 6 + 2; Parents of adolescents 5 + 2 / 12 | 22% | Children: 4.75; adolescents: 4.0; parents of children: 4.32; parents of adolescents:  3.18 / - / - | ↑ social abilities (n.s)^w^  ↓ Post-Event Processing (*)^w^ | SSQ-C/P  (0 / 12 /  26.07)^w^  PEPQ-R  (0 / 12 / 26.07)^w^ | PROCESS (Hayes, 2013) |
| van der  Zanden,  2014^x^  (NLD)  [66] | *N* = 244  young adults  (CES-D = 10 – 45) | 20.9 (2.2)  / 16 – 25 /  84.4 | depression/  CES-D  (0 / 12 / 24) | CBT-based online group course  (*Master*  *Your Mood*) (121)  vs.  WL(123) | (iii) | 6 / 6 | 20% | 3.2 (2.2) / - / - | ↑ control (*)  ↓ anxiety (*) | Mastery Scale  (0 / 12 / 24)  HADS-A  (0 / 12 / 24) | According to Baron and Kenny (1986);  path analysis  Mediation model  (MacKinnon, 2008) in Mplus (Muthén & Muthén, 2012) |
| Wade,  2017^y^  (USA)  [67] | *N* = 113^z^  children with moderate/severe early traumatic brain injury and their parents | 5.4 (2.2)  / 3 – 9/  39 | Behavioral problems/  ECBI  (0 / - / 13.04, 26.07) | Internet-based parent training:  I-InTERACT (39)  vs.  Express (36)  vs.  website with information (38) | (ii) | 10 + 4 / 26.07;  7 + 1 /  Finished before 26.07 | 19%^aa^ | I-InTERACT: 7.4 (2.7);  I-InTERACT Express: 4.8 (2.7) / per week 1.5 – 2 hours / - / | ↑ changes in parenting behavior (n.s.)^bb^ | DPICS  (0 / - / 13.04, 26.07) | PROCESS  (Hayes, 2013) |
| Wilksch,  2019^cc^  (AUS)  [69] | *N* = 316  women who seek help to improve their own body image | 20.8 (2.26)  / 18 – 25 /  100 | depression/  DASS  (0 / 10 / 26.07, 52.14) | Media Smart-  Targeted (122)^v^  vs.  e-mail with tips for a positive body image (194) | (i) | 9 / 9 | 32% | - /- / 13.6 % | ↓ eating disorder (*) | EDE-Q global  (- / 10 / 26.07, 52.14) | PROCESS  (Preacher & Hayes, 2004) |
| DHI_HP_ |  |  |  |  |  |  |  |  |  |  |  |
| Collins, 2002  (USA)  [48] | *N* = 100  students who report at least two episodes of heavy drinking in the last month  ( ≥ 5/4 alcoholic drinks; men/women) | 18.67 (1.02)  / - /  50 | Risky drinking behavior/  DNRF;  DDQ-M;  F-Q, RAPI  (0 / 6 / 26.07) | PNF via  email E-Mail (49)  vs.  psychoeducational brochure on alcohol (51) | (ii) | 1^dd^ / - | 6% | ‐ / - / - | ↓ discrepancy between own drinking behavior and that of others (n.s.) | Discrepancy  and  comfortratings^ee^  (0 / 6 / 26.07 ) | Baron and Kenny (1986); Holmbeck (1997) |
| Doumas, 2009  (USA)  [50] | *N* = 76^ff^  students who violated university regulations regarding alcohol and | 19.24 (1.33)  / 18 – 24 /  27.6 | Risky drinking behavior / drinking amount, maximum consumption, frequency of alcohol intoxication  (0 / - / 4.29) | Web-based PNF (*Try Our Free Drinking Evaluation*) (45)^ff^  vs.  web-based training (*Judicial Educator*) (31) | (ii) | 1^dd^ / 37.30 Min.^gg^;  1 / 49.19 Min.^gg^ | 12%^aa^ | ‐ / - / - | ↓ estimation of alcohol consumption by peers (*) | DDQ  (0 / - / 4.29) | According to Baron and Kenny (1986) |
| Doumas, 2011  (USA)  [51] | *N* = 135  students who violated university regulations regarding | 19.07 (1.01)  / 18 – 24 /  30 | Risky drinking behavior / weekly drinking amount, frequency of binge drinking, maximum alcohol consumption  (0 / - / 34.76^hh^) | Web-based feedback (*e-CHUG*):  Feedback without human support (54)  vs.  Feedback with human support (81) | (ii) | 1^dd^ / 39.0 Min.^gg^;  1^dd^ / 42.7 Min.^gg^ | 38%^aa^ | ‐ / - / - | ↓ estimation of alcohol consumption by peers (*) | Items to assess the drinking behavior of peers^ii^  (0, 34.76^hh^) | According to Baron and Kenny (1986); Sobel test (MacKinnon et al., 1995) |
| Dunn,  2019  (USA)  [52] | *N* = 134  students  (AUDIT > 7 and < 20; at least one episode of binge drinking in the last 30 days) | 19.42 (1.74)  / ‐ /  33 | Harmful alcohol consumption^ii^  ^jj^ / BYAACQ, seven items of the TLFB,  (0/ - / 4) | Web-based ECALC (58)  vs.  BMI with PNF(76) | (ii) | 1 / 45 Min.;  1 / 45-55 Min. | 18%^aa^ | ‐ / - / - | ↓ (positive) alcohol-related expectations (*) | CEOA  (0 /  Immediately following the intervention  /-) | In Mplus 8  (Muthén & Muthén, 2017;  MacKinnon, 2008) |
| Geisner, 2006  (USA)  [53] | *N* = 177  students with depressive (BDI-II ≥ 14) | 19.28 (1.97)  /‐ /  70 | depression/  BDI-II, DDS /  (0 / - / 4.34) | Compressed personalized feedback via email (89)  vs.  letter of appreciation (88) | (i) | 1^dd^ / - | 6%^aa^ | ‐ / - / - | ↓ hopelessness (*)  ↑ readiness for coping (n.s.) | HS  (0 /- / 4.35)  SHS  (0 / - / 4.35) | According to Baron and Kenny (1986);  McKinnon and Dwyer, (1993);  Sobel-Test (Sobel, 1982) |
| Gilmore, 2016 (USA)^kk^  [55] | *N* = 264  female students who report at least one episode of binge drinking within the last month weibliche | 18.77 (0.76)  / 18 – 21^ll^ /  100 | Binge drinking/ frequency of binge drinking episodes („How often did you have 4 or more drinks with alcohol within 2 hours?“)  (0 / - / 13.04) | Web-based feedback intervention: Complete (211)  (only alcohol vs. reducing the risk of sexual assault vs. combination vs. CG vs. minimal investigation) (53) | (i) | 1^dd^ / - | 22%^aa^ | ‐ / - / - | ↓ alcohol consumption as coping  (n.s.)^mm^ | DMQ-R SF  ( 0 / - / 13.04) | In Mplus 7 (Muthén &  Muthén, 1998-2011) |
| Jouriles, 2010  (USA)  [57] | *N* = 101  students who report more than one episode of heavy drinking in the last month  (≥5/4 alcoholic drinks; men/women) | 20 (2.0)  / ‐ /  79.59 | Risky drinking behavior / 14 days drinking calendar modified by DDQ  (0 / - / 2) | Web-based feedback intervention  (*e-CHUG*):  Reading feedback (30)  vs.  remember feedback  (36)  vs.  Typical (35) | (ii) | 1^dd^ / 20 Min. reading feedback;  20 Min. reading feedback;  20 Min.  Rewrite remembered informations; - | 3% | ‐ / - / - | ↑ remembered information (* partial for number of alcoholic drinks, most strongly with the most drinks; n.s. for total number of consumed alcoholic | number of remembered information  (- / - /2) | Path analysis, multiple regression  (MacKinnon, 2008) |
| Lewis,  2014  (USA)  [61] | *N* = 480  students who report one or more episodes of heavy drinking in the last month  (≥5/4 alcoholic drinks; men/women) | 20.38 (1.56)  / 18 – 25 /  57.6 | alcohol-related risky sexual behavior / measurement of drinking and sexual behavior^nn^ (0 / - / 13.04, 26.07) | web-based compressed personalized feedback: Only alcohol  (119)  vs.  alcohol-related risky sexual behavior (121)  vs.  combination (119)  vs.  feedback on media use (121) | (ii) | 1^dd^ / - | 10%^aa^ | Frequency of consideration of the feedback 2.18 (0.97) / duration of the first consideration of the feedback 1.27 Min (2.10) / participants who print out the feedback:  3.4 % | ↓ perceived norm: (*typical drinking quantity per week, frequency of alcohol consumption per month, typical drinking quantity per drinking situation, frequency of alcohol consumption before sex) | DNRF, items adapted by Lewis et al.  (Lewis et al., 2007)  (0 / - / 13.04, 26.07) | According to Baron and Kenny (1986) |
| Murphy,  2010^e^  (USA)  [62] | *N* = 133  students who report one or more episodes of heavy drinking in the last month  (≥5/4 alcoholic drinks; men/women)^oo^ | 18.6 (1.2)  / - /  50 | Risky drinking behavior/  DDQ  (0 / immediately following the intervention/ 4.35) | BMI via F2F (*BASICS*) (46)  vs.  web-based feedback intervention  (*e-CHUG*) (45)  vs.  Interview (42) | (i) | 1^dd^ / 50–60 Min.^gg^;  1^dd^ / 6 – 7 Min. assessment and 30 Min.  reviewing feedback  - | 9%^aa^ | ‐ / - / - | ↑ motivation to change drinking behavior (n.s  ↓ alcohol-related discrepancy (normative and personality ideal) (n.s.) | The Readiness Ladder  (0 /  Immediately following the intervention/ 4.35);  Discrepancy Ratings Questionnaire (0 /  Immediately following the intervention/ 4.35) | Not specified^qq^ |
| Neighbors, 2010  (USA)  [63] | *N* = 818  students who report having had one or more episodes of heavy drinking in the last month at baseline | 18.16 (0.6)^rr^  / - /  57.8^rr^ | Risky drinking behavior/  DDQ  (0 / - / 26.07, 52.14,78.21, 104.29) | Web-based feedback: gender-specific once  (163)  vs.  gender-specific semi-annual(164)  vs.  not gender- specific once (164)  vs.  not gender-specific semi-annual (163)  vs.  interview (164) | (i) | Feedback onetime: 1 / 5 assessments over 50 Min. (semi-annual)  Feedback semi-annual:  5 / 5 assessments over 50 Min (semi-annual) | 8%^aa^ | ‐ / - / - | ↓ gender-specific perceived norms (*)  ↓ gender-unspecific perceived norms (n.s.) | Modified DNRF (0 / - / 26.07, 52.14,78.21, 104.29),  DNRF (0 / - / 26.07, 52.14,78.21, 104.29), | According to Kenny et al. (2004) |
| Walters, 2007  (USA)  [68] | *N* = 106  Students, in the first semester, who report more than one episode of heavy alcohol consumption in the last month  (≥5/4 alcoholic drinks, men/women) | ‐  /‐/  48.1 | Risky drinking behavior/  DDQ, BAC, RAPI, seven alcohol-related questions^ss^  (0 / - / 8, 16) | Web-based feedback intervention  (*e-CHUG*):  Feedback afterwards (50)^p^  vs.  WL (56)^p^ | (ii) | 1^dd^ / - | 28%^aa^ | ‐ / - / - | ↓ estimation of the perceived drinking norm (*drinks per week, BAC) | Evaluation of one‘s own drinking behavior in relation to other U.S. students („How many U.S. students (in percent) drink more than you“)  (0 / - / 8, 16) | According to Baron and Kenny (1986) |
| Young,  2019  (USA)  [70] | *N* = 250  students who report more than one episode of heavy drinking in the last month (≥5/4 alcoholic drinks; men/women) | 21.02 (2.16) /  18 – 26 /  70.4 | Heavy alcohol consumption/ DDQ, RAPI, BYAACQ  (0/ - / 4.35, 26.07^tt^) | PNF (62)  vs.  PNF plus expressive writing (63)  vs.  expressive writing (63)  vs.  feedback on media use (62) | (i) | 1^dd^/ - ;  1^dd^ / 15-20 Min. expressive writing;  1 / 15-20 Min.  expressive writing; 1^dd^ / - | 32%^aa^ | ‐ / - / - | ↓ perceived drinking norm^uu^ | DNRF  (0/ - / 4.35) | Mplus 8 (no information about the authors) |

- = no data available; * = significant; ↑↓ = change in mediators according to the hypothesis; one year = 52.14 weeks; one month = 4.35 weeks; one day = 0.14 weeks; Min. = minutes; min.= at least; n.s. = not significant; AUS = Australia; GBR = Great Britain; NLD = The Netherlands; SWE = Sweden; USA = United States of America; ACT = Acceptance and commitment therapy; ADHS = attention deficit-hyperactivity disorder; ADIS-C/P = Anxiety Disorders Interview Schedule for Children and Parents; AUDIT = The alcohol use disorders identification test, BAC = peak blood alcohol concentration; BDI-II = Beck Depression Inventory-II; BFST-D = Behavioral Family Systems Therapy for Diabetes; BMI = Brief Motivational Intervention; BYAACQ = Brief Young Adult Alcohol Consequences Questionnaire; CBQ = Conflict Behavior Questionnaire-20; CCAPS-34 = The Counseling Center Assessment of Psychological Symptoms; CDI = Children's Depression Inventory; CEOA = Comprehensive Effects of Alcohol Scale; CES-D = Center for Epidemiologic Studies Depression Scale; CGAS = Children’s Global Assessment Scale; DASS (-21) = Depression Anxiety Stress Scale; DBAS = Dysfunctional Beliefs About Sleep Scale; DBD = Disruptive Behavior Disorders Rating Scale; DDQ (- M) = (modified modifiziert-) Daily Drinking Questionnaire; DDS = DSM-IV-Based Depression Scale; DFCS = Diabetes Family Conflict Scale; DMQ-R SF = Drinking Motives Questionnaire-Revised Short-Form; DNRF = Drinking Norms Rating Form; DPICS = Dyadic Parent–Child Interaction Coding System; ECALC = Expectancy Challenge Alcohol Literacy Curriculum; ECBI = Eyberg Child Behavior Inventory; EDE-Q global = Eating Disorder Examination Questionnaire; ESA-Scale = adapted by the 20 items Self-reflection and Insight Scale, the 10 items Ruminative Response Scale, and the 12 items Meta-Evaluation Scale; F2F = Face-to-face; FCU = Family Check-Up; F-Q = Frequency-quantity questionnaire (adapted by Borsari and Carey (Borsari & Carey, 2000) and Dimeff and colleagues (Dimeff, 1999)); GAD-Q-IV = Generalized Anxiety Disorder Questionnaire for DSM-IV; HADS-A = Hospital Anxiety and Depression Scale; HHI = The Helping for Health Inventory; HS = Hopelessness Scale; HSDQi = Holland sleep disorders questionnaire subscale insomnia Subskala Insomnie; I-InTERACT/ Express = Internet-based Interacting Together Everyday: Recovery After Childhood TBI; iCBT = internet-based cognitive behavioral therapy; K10 = Kessler Psychological Distress Scale; CBT = cognitive behavioral therapy; Mastery Scale = Pearlin Mastery Scale; MCQ = The Meta-Cognitions Questionnaire; MCTQ = Munich Chronotype questionnaire; PCI = Present Control Intervention; PCOSES = Perceived Control Over Stressful Events Scale (subscale Subskala); PEPQ-R = Post Event Processing Questionnaire Revised; PHLMS = Philadelphia Mindfulness Scale; PPI = Parenting Practices Inventory/Interview (subscales); PNF = personalized normative feedback; PSAS = Pre-sleep Arousal Scale; PSQI = Pittsburgh Sleep Quality Index; PSQI-A = Pittsburgh Sleep Quality Index Addendum; PSWQ = The Penn State Worry Questionnaire; RAPI = Rutgers Alcohol Problem Index; SCI = Sleep condition indicator; SDQ = Strengths and Difficulties Questionnaire; SHS = The Self-Help Scale; SPAI-C = Social Phobia and Anxiety Inventory for Children; SSQ-C/P = Social Skills Questionnaire - Child and Parent Versions; TL = Thought Log; TLFB = Alcohol Timeline Followback Method (seven items: MBAC = mean blood alcohol concentration; PBAC = peak blood alcohol concentration; MDPS = mean drinks per sitting; PDPS = peak drinks per sitting; MDPW = mean drinks per week; DDPM = drinking days per months; Binge = Number of binge episodes); VQ = Valuing Questionnaire; WL = waitlist-condition; WOJ = Worry Outcome Journal; YSR = Youth Self-Report; ^a^ mental disorder = disorder/symptoms addressed by the intervention and included by the authors in the mediation analysis; transdiagnostic = intervention addresses multiple disorders. Differed disorders are considered simultaneously^b^; MTP = measurement time points: Baseline, Post-Treatment, Follow-Up(s); ^c^ classification according to Berger and colleagues (Berger, 2017): (i) = without human support („web-based unguided self-help programs”), (ii) = with minimal human support („internet-based guided self-help approaches”), (iii) = the internet as the sole means of communication („internet-based psychotherapies”); ^d^ The study drop-out rate was calculated as the quotient of the number of participants who finished the intervention (post-treatment) and the number of randomized participants (*N*) substracted from 100%; ^e^ Data from study 2 were extracted; ^f^ Data from adolescents (12 to 18 years) were included in the mediation analysis; predictor mediation analysis *working alliance;* ^g^ Data refer to the EG. Data from the CG were collected for MTP 0, 8.7. The participants in the CG started treatment after 8.7 weeks.; ^h^ The mediation anaylsis was carried out with the data up to MTP 8.7, as the data from both the EG and the CG were available up to this point.; ^i^ information can originate from Denis and colleagues (Denis et al., 2017), among others; ^j^ Data originates from table 3 *„*participations rates and adherence“; ^k^ Due to the high drop-out rate, data from the follow-up assessment were not included in the analysis; the analyses were only carried out with data from post-treatment.; ^l^ Derived from information in the text: If participants didn’t complete the module in the given time, the next module was sent within the following 2-3 days.; ^n^ calculated on the basis of *N* = 114; ^o^ information can originate from LaFreniere and Newman (LaFreniere & Newman, 2016), among others; ^p^ information was provided by the author upon request.; ^q^ predictor mediation analyis *uncontrollability of beliefs*; ^r^ Both mediators show significant effects for distress, depression, generalized anxiety disorder, phobias, social phobias. In addition, the mediator *obstruction of appreciation of life* in relation to academic worries was significant.; ^s^ Information can originate from Harris et al. (2015) (Harris et al., 2015), among others.; ^t^ Information from the table was used in the case of different specifications.; ^u^ As there was no significant difference between groups, they were combined for the analysis.; ^v^ intervention is not explicitly aimed at the disorder; ^w^ Due to missing follow-up data in the WL, mediation analysis was based only on data from baseline and 12-week post-treatment (children/adolescents or their parents who completed at least 3 sessions and provided data at 12-week post-treatment). Due to this limitation, the authors were not able to draw a conclusion regarding causality, as a test of longitudinal mediation was not possible.; ^x^ Information may originate from van der Zanden and colleagues (van der Zanden et al., 2012), among others; ^y^ Information can originate from the study by Narad and colleagues (Narad et al., 2017), among others.; ^z^ *N* = 117 participants were randomized. However, *n* = 3 participants did not complete the baseline measure and *n* = 1 person was subsequently excluded. Therefore, *n* = 113 participants took part in the intervention.; ^aa^ Calculation of the drop-out rate was based on data from the first follow-up assessment; ^bb^ Separate moderated mediation: Moderator had higher scores on ECBI (Baseline).; ^cc^ Information can originate from the study by Wilksch and colleagues (Wilksch et al., 2018), among others.; ^dd^ After data collection, participants received feedback.; ^ee^ Rating was developed specifically for these studies and consists of six items that assess the discrepancy between one’s own drinking behavior and that of others.; ^ff^ Originally, there were *N* = 46 participants in the *web-based PNF* group and *N* = 31 in the *web-based training* group. However, one person was excluded because there were technical problems in submitting the feedback. Therefore, data from *N* = 76 participants were reported.; ^gg^ Average duration of the entire appointment.; ^hh^ Average length between baseline and follow-up assessment.; ^ii^  DNRF, Estimate of frequency of binge drinking, estimate of highest number of drinks at a drinking occasion in the last month; ^jj^ includes alcohol-related harm and alcohol consumption (defined by seven variables of the TLFB); ^kk^ Information can also originate from Gilmore and colleagues (Gilmore et al., 2015); ^ll^ Original inclusion criteria 18 to 20 years.; ^mm^  In regard to the interventions *alcohol only* and *reducing the risk for sexual assaults* (n.s.). For the combined intervention in relation to binge drinking for participants with a higher motive for alcohol consumption as coping at baseline (*); ^nn^ A total of six outcome variables (alcohol-related consequences, frequency of drinking before sex, frequency of drinking per month, typical drinking amount per drinking situation, typical drinking amount per week) assessed by DDQ, Quantity/Frequency/Peak Alcohol Use Index, BYAACQ and items adapted by Lewis and colleagues (Lewis et al., 2007); ^oo^ Application of different criteria for minorities; ^qq^ In study 2, no information was provided on the statistical analysis; ^rr^ Data was based on the number of people who completed the screening (*N* = 2095). No complete details provided on the final randomized participants; ^ss^ The exact questions were not specified. Questions were based on, for example: AUDIT, risk of alcoholism, importance, amount of money spent on alcohol; ^tt^ Follow-up data (26.07) was not reported due to the high drop-out rate; ^uu^ (*) for personalized normative feedback, personalized normative feedback additionally expressive writing feedback.
