# Supplementary material for: Mechanisms of Change in Digital Health Interventions for Mental Disorders in Youth: Systematic Review
Source: J Med Internet Res. 2021 Nov 26;23(11):e29742. doi: 10.2196/29742 (PMC8665396; doi:10.2196/29742)
Supplement: Multimedia Appendix 3 [file jmir_v23i11e29742_app3.docx]

**Multimedia Appendix 3.**

Mechanisms of Change in Digital Health Interventions for Mental Disorders in Youth: Systematic Review

#

*Multimedia Appendix 3. Figure S.1 Studies with number of criteria met.*
